# Development of the Well-being questionnaire short-form in Japanese: the W-BQ12

**DOI:** 10.1186/1477-7525-4-40

**Published:** 2006-07-03

**Authors:** Afsane Riazi, Clare Bradley, Shalleen Barendse, Hitoshi Ishii

**Affiliations:** 1 Department of Psychology, Royal Holloway, University of London, Egham, Surrey, UK; 2 Diabetes Centre, Tenri Hospital, Nara, Japan

## Abstract

**Background:**

The Well-being Questionnaire (W-BQ) was designed to measure psychological well-being in people with diabetes. This study aimed to develop a Japanese version and a short form of the W-BQ.

**Methods:**

A linguistic validation process produced a preliminary Japanese version of the 22-item W-BQ, which was distributed to 550 patients. Factor structure, reliability (Cronbach's alpha) and aspects of validity (hypothesised group differences and correlations with other measures) were evaluated.

**Results:**

Questionnaires were returned by 464 patients (84.4%). Preliminary factor analysis revealed that the Depression and Anxiety items were dispersed according to the positive or negative direction of the wording. A 12-item W-BQ (Japanese W-BQ12), consisting of three 4-item subscales (Negative Well-being, Energy and Positive Well-being), was constructed that balanced positively and negatively worded items. Cronbach's alpha was high (>0.85) for the 12-item questionnaire and consistently high (>0.82) across sex and treatment subgroups. Cronbach's alpha for subscale scores in the total sample ranged from 0.69 (Energy) to 0.80 (Positive Well-being). Expected subgroup differences indicated significantly poorer well-being in women compared with men and in insulin-treated patients compared with tablet/diet treated patients. Discriminant and convergent validity was supported by minimal correlations between W-BQ12 scores and HbA1c and low-to-moderate correlations with Diabetes Treatment Satisfaction Questionnaire (DTSQ) scores.

**Conclusion:**

The W-BQ12 (Japanese) is a short, reliable and valid measure of psychological well-being that is suitable for use with people with diabetes. The items selected to produce the W-BQ12 (Japanese) have since produced psychometrically sound 12-item short-form measures in other translations for use in diabetes and in other chronic illnesses.

## Background

There are several reasons why it is important to measure psychological outcomes in diabetes care. First, psychological outcomes are important in their own right and they need to be monitored if they are to be optimised [1]. Secondly, it is important to ensure that improved metabolic control is not achieved at the expense of psychological outcomes. Improved metabolic control may contribute to improved psychological well-being and vice versa, but a positive correlation cannot be assumed [2].

The 22-item Well-being Questionnaire (W-BQ22) [3,4] was originally designed in 1982 for use in a World Health Organisation study evaluating new treatments for the management of diabetes. It consists of four subscales measuring: Depression, Anxiety, Energy and Positive Well-being. A total well-being score can also be calculated by combining the subscales. The depression and anxiety subscales were derived in earlier work by Warr et al [5] using items originating from Zung scales [6].

W-BQ22 items focus on cognitive symptoms of mood states. Items concerning somatic states were avoided as they may lead to criterion contamination in populations with diabetes, where somatic symptoms such as fatigue or loss of appetite (common symptoms of depression in the general population) may be due to the physical condition of diabetes rather than depression. The W-BQ22 has been linguistically validated into many languages and has been recommended for use by the World Health Organisation/International Diabetes Federation [1]. The W-BQ22 contains no overtly diabetes-specific questions and it has been found to work well in adults with other chronic conditions including growth hormone deficiency [7] and macular disease [8].

The aim of the study was to develop a Japanese version of the W-BQ suitable for use in diabetes research and clinical practice. The present paper reports the linguistic validation of the W-BQ into Japanese and subsequent psychometric development of the W-BQ12 short form.

## Methods

### Linguistic validation

A native Japanese bilingual health psychologist (AR) conducted the initial translation of the W-BQ22 into Japanese. Optional translations of some of the items were produced with a view to selecting the most appropriate translation following back-translation, clinician review and cognitive debriefing with patients. Additional items were also translated into Japanese in anticipation that some of the concepts of psychological well-being would not travel well between Europe and Japan. Five additional items were selected from the original Zung questionnaire [6] and evaluated along with the 22 items. Three items were selected as candidates for the Depression subscale ('I feel hopeful about the future', 'If I am gone, other people's lives would benefit', and 'Everything will be fine and nothing bad will happen'), and two for the Anxiety subscale ('I feel more irritated than usual' and 'I have nightmares'). Back-translation of all 27 Japanese items (including optional translation alternatives) into English was conducted by two native British translators, fluent in Japanese. Where there were discrepancies between the backtranslations and original English, items were reviewed and discussed with the translators and with the consultant physician at Tenri Hospital (HI). The most appropriate translations were then selected from the options available for all 27 possible items. The resulting draft questionnaire was then pre-tested in cognitive debriefing interviews with eight patients attending the diabetes clinic at Tenri Hospital to establish whether patients' understanding of each item was as intended [9], and the final selection of translation options was made.

### Patients and procedures

A questionnaire booklet containing the Japanese 27-item W-BQ, the Japanese Diabetes Treatment Satisfaction Questionnaire (DTSQ; [10]), and demographic questions was distributed to 550 consecutive patients attending the out-patient clinic of Tenri Hospital. During pilot-testing, it became apparent that a considerable proportion of patients attending the Tenri Hospital were people with affiliation to Tenri-kyo (a minority religion in Japan). Thus the demographic questions included a question regarding the patient's religious affiliations. Furthermore, patients were asked to provide their doctor's name if they wished their doctor to see their responses to the questionnaires. This procedure was adopted to ensure that patients understood that their responses would not otherwise be seen by their doctor and to provide an opportunity for those who particularly wanted their doctor to see their questionnaires to let that be known. Completed questionnaires were returned to the hospital clinic, and were then forwarded, unopened, to the Diabetes Research Group at Royal Holloway, University of London. When patients expressed a wish for their doctor to see their responses, a copy of the questionnaire was sent to the doctor. The ethics committee of the Tenri Hospital approved the study.

The HbA1 levels (normal range 5.7–8.0) for each patient were identified from medical records. Questions relating to hypoglycaemia were also included with the demographic questions. The *frequency of hypoglycaemia score *was calculated from responses to the question: 'In the past two months, how many times have you experienced symptoms of hypoglycaemia? '. The *severity of hypoglycaemia score *was calculated by summing the responses to the following questions: *'How many times have you lost consciousness because of hypoglycaemia at any time in the past two months?'*, *'How many times have you in the past two months experienced hypoglycaemia without losing consciousness but still needed someone's help to recover from the episode?' and 'How many times have you in the past two months felt too ill to go to work or follow your usual daily routine because of hypoglycaemia?*'. The frequency and severity scores were multiplied to obtain a *severity × frequency *score. Thus for all three scores, the higher the score, the higher the impact of hypoglycaemia was likely to be on the participants' lives.

### Statistics

The preliminary scale structure was evaluated by principal components analysis with Oblimin rotation, chosen because previous work suggested that the factors would intercorrelate [3]. However, principal components analysis with Varimax rotation was also conducted for comparison purposes in order to minimise correlations between components. Reliability was evaluated by Cronbach's alpha coefficients of internal consistency [11]. The preliminary scale was shortened to 12 items by eliminating items with the least favourable psychometric properties (lower factor loadings and lesser contributions to the internal consistency of the relevant subscale to achieve a well-balanced questionnaire with equal numbers of positively and negatively worded items and subscales of equal length).

Factor analysis and reliability analyses were repeated on the resulting 12-item version (Japanese W-BQ12). These analyses were also repeated for the two sexes, major treatment subgroups (insulin-, tablet-, and diet-treated), religious groups, and those who did and did not want their doctor to see their responses, in order to investigate whether the structure or the reliability of the scale differed for different subgroups.

Group differences validity was evaluated by examining the W-BQ12 scores and its subscale scores for groups expected to differ in a predictable way. Based on previous work with the W-BQ22 [e.g. [3]], it was expected that: women would score higher than men on the Negative Well-being subscale, indicating more depression/anxiety in women; insulin-treated patients would show impaired well-being compared with tablet- and/or-diet-treated groups, and insulin-treated patients with complications of diabetes would show impaired well-being compared with insulin-treated patients without complications. Convergent and discriminant validity were determined by examining the extent to which correlations between W-BQ12 and other measures (HbA1, measures of hypoglycaemia and DTSQ scores of treatment satisfaction) were consistent with predictions. Minimal correlations (*r *< 0.30) between W-BQ-12 subscales and HbA1c were expected. Low-to-moderate correlations (*r *= 0.30 - 0.60) between the W-BQ12 and measures of hypoglycaemia and DTSQ scores were expected. It was expected that more frequent and/or severe hypoglycaemia would be associated with reduced well-being, and greater satisfaction with treatment would be associated with greater well-being.

Non-parametric Kruskal Wallis tests for group comparisons (providing Chi-Squared statistics) and Spearman correlations (*r*s) were used to take account of the skew in W-BQ scores which in subgroup analyses of smaller sample sizes could mislead if parametric tests had been used.

## Results

### Linguistic validation

The Japanese translation captured the content of the original W-BQ22 with appropriate adaptations to several words where an equivalent Japanese word for the original English did not exist. For example, the Anxiety item 'I feel nervous and anxious' and the Energy item 'I feel tired, worn out, used up, or exhausted' required additional words in the Japanese translation to capture the breadth of meaning of the English original. On the other hand, the Energy item 'I feel energetic, active or vigorous' used only two Japanese words rather than three, as the two Japanese words captured the meaning of all three English words.

The backward translations were similar to the original English version of the W-BQ22. Any minor differences were in the choice of words of a similar meaning. Minor adjustments were made to wording of some items following the backtranslation. No further changes needed to be made following cognitive debriefing with patients to pilot test the instrument. The five additional items were generated as optional supplementary items in case the understanding of the original items was found to be problematic during cognitive debriefing. As there were no problems in the understanding of the original 22 items, the five additional items were not used in the analysis.

### Psychometric evaluation of the Japanese W-BQ

#### Sample

Four-hundred and sixty-four (84.4%) patients returned the questionnaires. This sample provided sufficient numbers of insulin-treated, tablet-treated and diet-alone-treated patients for subgroup analyses (Table 1). HbA1 levels were available for 425 of the 464 participants (91.6%).

**Table 1:** Sample characteristics

Variable	Total sample
N	464
Gender	
Women (%)	48
Age	
Mean (sd)	58.7 (12.4)
Range	19 – 90
Years since diagnosis	
Mean (sd)	9.9 (8.0)
Range	0.5 – 40
Marital status	
Married (%)	96
Employment status	
Employed (%)	46
Treatment	
Insulin (%)	41
Tablet (%)	40
Diet only (%)	19
Diabetes complications	
Yes (%)	43
Illness apart from diabetes	
Yes (%)	35
HbA1	
Mean (sd)	7.0 (1.3)
Range	5–13
Religious affiliation	
Tenri (%)	33

### Factor analysis of the Japanese W-BQ

Unforced principal components analysis of the 22-item Japanese translated version of the W-BQ with Oblimin rotation provided four factors with eigenvalues greater than 1. Although positive well-being items and energy items loaded appropriately on separate factors, items for the depression and anxiety subscales were dispersed across the first 3 factors with energy items characterising the fourth factor. Because a clear four-factor solution was not seen, a forced three-factor solution was undertaken to see whether the depression and anxiety items would load together on one factor. In a forced three-factor solution, the four positively worded depression items loaded on factor 1 along with all six of the positive well-being items. The two negatively-worded depression items loaded on factor 2 along with the four negatively worded anxiety items. The two positively worded anxiety items loaded on factor 3 with the energy items. The effect of positive versus negative wording has been found in past datasets to create some overlap between depression and anxiety items [3]. Very similar patterns of loadings were obtained with the Varimax rotation. Thus, the first factor was the positive well-being items with the positively worded depression items included, the second factor was negative well-being including negatively worded anxiety and depression items, and the third factor was energy with the positively worded anxiety items included.

### Development of the Japanese W-BQ12

One way in which the W-BQ might be improved in general, not only in the Japanese version, is to balance the numbers of positively and negatively worded items both within the subscales and across the scale as a whole. The W-BQ22 consists of an overall preponderance of positively worded items (14 positively worded to 8 negatively worded), and the proportions vary within the subscales (Depression scale, 4 to 2, Anxiety, 2 to 4, Energy, 2 to 2, and Positive Well-being, all 6 positively worded). Thus, in order to overcome the problem of the Depression and Anxiety subscales splitting according to the positive or negative direction of the wording, negatively worded Depression, Anxiety, and optional additional items were examined with a view to creating a Negative Well-being subscale made up entirely of negatively worded items. Such a subscale would complement the Positive Well-being subscale that consists only of positively worded items. Energy and Positive Well-being items were also examined with a view to reducing the number of items to keep the length of the questionnaire to a minimum. The following issues were considered in developing the short-form: the balance of positive and negatively worded items across the scale and within subscales, content of the items (ensuring that the items cover the breadth of meaning of the construct being measured), Cronbach's alpha, and finally, factor loadings on the three-factor solution.

#### i) Depression

There were only two negatively worded Depression items and both of these were retained.

#### ii) Anxiety

In order to maintain the balance between Depression and Anxiety items in the subscale, two negatively worded anxiety items were selected on the basis of reliability and factor loadings.

#### iii) Positive well-being

In the interests of balance, two items were selected for exclusion. Factor loadings were high for all six items (>= 0.68) so items to be excluded were selected on grounds of reliability and content (to ensure that the breadth of content was retained). Items that contributed the most to the Cronbach's alpha and the items that best captured the breadth of content of the construct of positive well-being were retained.

#### iv) Energy

The four-item Energy subscale had an alpha coefficient of 0.69 and exclusion of any item reduced the reliability. All four items were retained.

The resulting 12-item Well-being Questionnaire was balanced for positively and negatively worded items (six of each). All subscales consisted of four items. Only the Energy subscale included a mix of positive and negatively worded items (2 of each).

### Factor analyses of the Japanese W-BQ12

The factor structure of the Japanese W-BQ12 is shown in Table 2. Factor 1 includes all the Positive Well-being items together with overlap from the two positively worded Energy items. Factor 2 includes all four Negative Well-being items together with slight overlap from one of the Positive Well-being items loading negatively on this factor (though not as highly as it loads on Factor 1 with the other Positive Well-being items). There was also slight overlap from a negatively worded Energy item though it loaded far more strongly on the third factor. Factor 3 includes only the Energy subscale items with no overlap from other items. All but two items (both Energy items) showed loadings well in excess of 0.4 indicating well-defined factors. A forced one-factor solution confirmed that all 12 items loaded highly (range 0.539 – 0.700) on the same factor. Using the Varimax rotation, very similar factor loadings of the Japanese W-BQ12 were obtained for the three-factor solution.

**Table 2:** W-BQ12 (Japanese) items and factor analysis rotated component matrix

**Subscale item**	**Item**	**Factor**		
		
		**1**	**2**	**3**
Neg 1	I have crying spells or feel like it	-0.263	**0.769**	-0.214
Neg 2	I feel downhearted and blue	-0.291	**0.736**	-0.279
Neg 3	I feel afraid for no reason at all	-0.271	**0.829**	-0.278
Neg 4	I get upset easily or feel panicky	-0.242	**0.757**	-0.356
Energy 1	I feel energetic, active or vigorous	0.667	-0.065	0.361
Energy 2 (reversed)	I feel dull or sluggish	0.310	-0.386	**0.887**
Energy 3 (reversed)	I feel tired, worn out, used up or exhausted	0.270	-0.400	**0.891**
Energy 4	I have been waking up feeling fresh and rested	0.668	-0.242	**0.406**
Pos 1	I have been happy, satisfied or pleased with my personal life	**0.768**	-0.306	0.147
Pos 2	I have lived the kind of life I wanted to	**0.755**	-0.435	0.121
Pos 3	I have felt eager to tackle my daily tasks or make new decisions	**0.818**	-0.197	0.191
Pos 4	I have felt I could easily handle or cope with any serious problem or major change in my life	**0.755**	-0.298	0.160

The factor structure found in the total sample was replicated within the subgroups of patients treated with insulin and those treated with tablets. However, in the diet-alone treated subgroup a 2-factor solution emerged. All four Energy items loaded >0.4 with the Positive Well-being items and the two negatively-worded Energy items loaded even more highly with the Negative Well-being items on the second factor. These findings support the observation that the two primary dimensions of mood, Positive Affect (PA) and Negative Affect (NA) are not opposites of each other, but are two highly distinct dimensions that are represented as orthogonal dimensions [12].

The factor structure within the subgroups of men and women was replicated satisfactorily. However, for the men, the Energy item 'I feel energetic active or vigorous' loaded in excess of 0.7 with the Positive Well-being items and loaded less than 0.36 with the other energy items on factor 3. For the women, this Energy item loaded in excess of 0.4 with the other Energy items, but the Energy item 'I have been waking up feeling fresh and rested', loaded in excess of 0.7 with the Positive Well-being items, while having a loading less than 0.29 with the other Energy items. These findings suggest some sex differences in the forms of energy that were associated with positive well-being in this Japanese sample. Feeling energetic appeared to be more important for the positive well-being of the men while feeling rested seemed to be more important for the positive well-being of the women.

When the sample was split by patients who wanted their doctor to see their responses and patients who did not want their doctor to see their responses, or into the two main religious groupings of interest, Tenri versus other religions, the factor structure replicated well with no anomalies (data not shown).

### Reliability analyses of the Japanese W-BQ12

Internal consistency estimates were highly satisfactory for the four-item subscales (Table 3). All alpha coefficients exceeded the recommended criterion of 0.7 [11] except for Energy (0.69). Alpha for the total scale was 0.85. These results were consistent within the subgroups broken down by sex and by treatment groups, and were similar to estimates obtained for the 22-item English version for samples of patients with type 1 and those with type 2 diabetes [3].

**Table 3:** W-BQ12 (Japanese) subscales: Cronbach's alpha and item-total correlations

**Subscale item**	**Alpha**	**Corrected item-total correlation**	**Alpha if item deleted**
**Negative Well-being**	0.78		
Neg 1		.5606	.7286
Neg2		.5599	.7358
Neg 3		.6327	.6934
Neg 4		.5708	.7229
			
**Energy**	0.69		
Energy 1		.3515	.7021
Energy 2 (reversed)		.5816	.5573
Energy 3 (reversed)		.5518	.5782
Energy 4		.4303	.6566
			
**Positive Well-being**	0.80		
Pos 1		.5942	.7665
Pos 2		.6144	.7570
Pos 3		.6577	.7362
Pos 4		.6094	.7597

### Scoring of the Japanese W-BQ12

Negative Well-being subscale items and Positive Well-being subscale items are summed to produce two subscale scores (range 0 – 12) where a higher score reflects more negative or positive well-being respectively. Energy subscale items can be summed after reversing the scores of the two negatively worded items to produce a subscale score (range 0 – 12) where a higher score indicates more energy. The formula used to calculate total General Well-being from all 12 items (range 0–36) is: 12 - Negative Well-being + Energy + Positive Well-being.

### Validity of the Japanese W-BQ12

Construct validity (Table 4)

**Table 4:** W-BQ12 (Japanese) and subscales: subgroup mean scores, standard deviations and Cronbach's alpha

	Treatment groups				Sex			Total Sample
	
	Insulin	Tablet	Diet	*χ*^2 ^(*df*)	Men	Women	*χ*^2 ^(*df*)	
General Well-being (total)								
Mean (sd) [*χ*^2 ^(*df*)]	25.1 (6.3)	26.9 (6.2)	27.3 (5.4)	[10.7 (2)**]	26.6 (6.1)	25.8 (6.3)	[1.6 (1)]	26.2 (6.2)
alpha	0.84	0.87	0.82		0.87	0.84		0.85
Negative Well-being								
Mean (sd) [*χ*^2 ^(*df*)]	2.8 (2.8)	2.0 (2.3)	2.1 (2.3)	[7.5 (2)*]	2.0 (2.4)	2.7 (2.7)	[8.6 (1)**]	2.3 (2.6)
alpha	0.81	0.73	0.77		0.79	0.76		0.78
Energy								
Mean (sd) [*χ*^2 ^(*df*)]	7.3 (2.6)	8.0 (2.6)	8.4 (2.4)	[11.2 (2)*]	7.7 (2.6)	7.9 (2.6)	[0.4 (1)]	7.8 (2.6)
alpha	0.68	0.70	0.70		0.72	0.65		0.69
Positive Well-being								
Mean (sd) [*χ*^2 ^(*df*)]	8.5 (2.5)	8.9 (2.6)	8.9 (2.3)	[2.4 (2)]	8.8 (2.4)	8.6 (2.6)	[0.3 (1)]	8.7 (2.5)
alpha	0.77	0.84	0.80		0.82	0.79		0.80

*p < 0.05; **p < 0.01

As expected from use of the W-BQ22 in other languages and from other measures of depression and anxiety, the mean score of the Negative Well-being subscale was significantly higher in women, indicating more negative well-being in women than in men. Insulin-treated patients reported higher Negative Well-being, lower Energy, and less total General Well-being than tablet- or diet-treated patients. However, the significant effect of treatment group disappeared when measures of hypoglycaemia experienced were controlled for. Thus the correlation between treatment group and General Well-being score was (*r *= -0.15, n = 411, p = 0.002) but when recent experience of hypoglycaemia (regardless of frequency or severity) was partialled out of the correlation the association between well-being and treatment disappeared (*r *= 0.07, n = 407, p = 0.16). Insulin-treated patients who had complications of diabetes had worse Negative Well-being, Energy, and total General Well-being than insulin-treated patients who had no complications of diabetes. There were no differences between the well-being scores of those with complications and those without in the tablet and/or diet treated groups.

### Relationships between well-being and diabetes control (Table 5)

**Table 5:** Correlations between W-BQ12 (Japanese) scales and diabetes control and DTSQ for the total sample

	W-BQ12 (Japanese)			
	
	Negative Well-being	Energy	Positive Well-being	General Well-being (total)
Diabetes control				
HbA1^1^	0.01	-0.04	0.01	-0.02
Frequency of hypoglycaemia	0.17**	-0.11*	-0.11*	-0.16**
Severity of hypoglycaemia	0.18**	-0.17**	-0.14**	-0.20**
Severity × frequency of hypoglycaemia	0.19**	-0.17**	-0.15**	-0.21**
				
DTSQ				
Treatment Satisfaction	-0.25**	0.32**	0.35**	0.38**
Perceived Hyperglycaemia	0.22**	-0.31**	-0.13**	-0.28**
Perceived Hypoglycaemia	0.30**	-0.25**	-0.12*	-0.29**

^1^Maximum N = 425; *p < 0.05; **p < 0.01; DTSQ = Diabetes Treatment Satisfaction Questionnaire Treatment Satisfaction = items 1, 4, 5, 6, 7 and 8 summed where a higher score = greater satisfaction; Perceived Hyperglycaemia where a higher score = greater perceived frequency of hyperglycaemia; Perceived Hypoglycaemia where a higher score = a greater perceived frequency of hypoglycaemia.

As expected, the correlations between the subscale scores and the total score of the W-BQ12 with HbA1 were minimal. This was consistent even when the analyses were conducted separately for the two sexes and for the three treatment groups. There were minimal correlations between General Well-being scores and HbA1 within the subsample of insulin-treated patients (*r *= -0.08; n = 159; p = 0.346), tablet-treated patients (*r *= 0.10; n = 148; p = 0.237) and diet-alone treated patients (*r *= 0.05; n = 68; p = 0.668).

Well-being scores correlated with measures of the frequency and severity of hypoglycaemia in the direction expected (more frequent and severe hypoglycaemia being associated with reduced well-being). The correlations were strongest between measures of the severity and severity × frequency measures and General Well-being (total) scores.

### Relationships between well-being and DTSQ scores (Table 5)

The correlations between well-being scores and DTSQ scale scores were low-to-moderate as expected. The DTSQ treatment satisfaction score correlated most strongly with the General Well-being score and least with the Negative Well-being subscale score though correlations had p values < 0.01 at both extremes. The item on the DTSQ measuring perceived frequency of hypoglycaemia correlated most strongly with Negative Well-being and least with Positive Well-being. An item on the DTSQ measuring perceived frequency of hyperglycaemia correlated the most with Energy and least with Positive Well-being.

### Comparison of W-BQ12 (Japanese) with W-BQ (English) (Table 6)

**Table 6:** Mean W-BQ Scores for Sample Groups: Comparison of W-BQ12 (Japanese) with W-BQ (English)

	Current sample (12 item) Adjusted	Sample 1^1^: Type 2 (18 item)	Sample 2^1^: Type 1 (22 item)
Depression	4.0^2^	3.2	3.7
Anxiety	2.9^2^	4.5	2.1
Energy	7.8	Not scored	8.1
Positive Well-being	13.1	13.2	12.9
General Well-being	50.0^3^	41.4	51.2

^1^From Bradley (1994)

^2^Scores adjusted to be equivalent to those obtained on the 6-item scale from the W-BQ22 and W-BQ18

^3^General Well-being score adjusted to be equivalent to the W-BQ22 General Well-being score

The Japanese W-BQ12 mean scale scores were adjusted to allow direct comparison with the W-BQ mean scores reported elsewhere [3]. Sample 1 was 239 patients with Type 2 diabetes treated with oral hypoglycaemic agents who participated in a study evaluating management of Type 2 diabetes [4]. Sample 2 was from people with Type 1 diabetes participating in a World Health Organisation study of continuous subcutaneous insulin infusion (CSII) pumps [14]. Scores from the two W-BQ12 Depression items were multiplied by 3 to be equivalent to the 6-item W-BQ22 Depression subscale. A similar transformation was made for the Anxiety items. The Energy subscales were the same and can be compared directly where available. The total General Well-being scores for the W-BQ12 were divided by 12 and multiplied by 22. The means from the present combined sample of insulin, tablet, and/or diet-treated patients fall, as expected, very close to or in between the means for an earlier insulin-treated sample and those for a tablet-treated sample. Where the means from the two samples were very similar, the means from the present combined sample did not fall in between the means of the two previous samples, but instead also had very similar means to the other two samples.

## Discussion

The linguistic validation of and psychometric development work on the Japanese version of the W-BQ22 led to the creation of the W-BQ12. The scale consists of three subscales (Negative Well-being, Energy and Positive Well-being) of equal length, and achieved a balance of positively and negatively worded items. This has improved the structure of the original W-BQ22 as well as providing a welcome short form.

The analysis of the factor structure of the scale demonstrated a small amount of overlap, with the two positively worded Energy items loading on factor 1 as well as factor 3. The same overlap has since been demonstrated in another dataset [8], and appears to be due to the fact that the Energy items have a propensity to load together but the two positively worded Energy items also tend to load with other positively worded items and the two negatively worded Energy items have a tendency to load with other negatively worded items [15]. Thus double loadings can occur. A forced one-factor solution confirmed that all items loaded highly (>0.54) on the same factor if required and provided support for combining all items into a single total General Well-being score.

The factor structure found in the total sample was similar within the two sexes, and within the insulin- and tablet-treated patients. However, there was a slight difference between men and women in the pattern of use of the two positively worded Energy items. Although the possibility of eliminating these two items was considered, it was decided that it would be better to tolerate this difference rather than disturbing the balance of positive/negative items by shortening the Energy scale. The factor structure found in the total sample was not replicated within the diet alone-treated group, but this may be explained by the smaller sample size of this particular subgroup (N = 70).

Highly satisfactory Cronbach's alpha coefficients were obtained for the total scale, demonstrating good internal consistency, and subscale alphas were satisfactory. Although the factor analyses indicated some small variation in structure between subgroups, internal consistency remained virtually unchanged within the two sexes and the three treatment subgroups, providing support for the reliability of the subscales.

The internal structure of the measure has since been found to be similar in a Dutch sample of people with diabetes [16], and in English samples of people with other chronic conditions, macular disease [8] and growth hormone deficiency [7], with Positive Well-being items loading highly on the first factor, accounting for the greatest proportion of the variance, Negative Well-being items loading on the second factor and Energy items loading on the third factor. This suggests that the subscale constructs account for similar proportions of the variance regardless of the translation used or population studied.

Evidence of construct validity was found in the scale's sensitivity to expected subgroup differences. Women reported significantly higher Negative Well-being than men as reported elsewhere [3]. That women show higher levels of anxiety and depression than men is well documented [17]. Insulin-treated patients reported worse Negative Well-being, Energy, and total General Well-being than tablet-or diet-treated patients. Further analyses suggested that the reduced Negative Well-being among insulin-treated patients may be entirely attributable to the experience of hypoglycaemia in this treatment group. Furthermore, insulin-treated patients with complications had worse well-being than those without complications. However, there were no differences between the well-being scores for those with complications and those without in the tablet-and diet- treated groups. This may be explained by the fact that in these older groups of patients, the presence of other illnesses unrelated to diabetes is likely to dilute any differences in well-being attributable to complications.

The factor structure and reliability of eight translations of the W-BQ12 (English, French, German, Dutch, Danish, Norwegian, Swedish and Finnish) have been shown to be excellent for all but Dutch in which further investigation with a larger sample size was needed [18]. A Dutch group have independently reported that the Dutch translation of the W-BQ12 demonstrated a clear 3-factor structure [16] as well as having good evidence of reliability and validity [19]. Thus, it appears that the selection of items made to produce the W-BQ12 (Japanese) is also producing a psychometrically sound instrument in other translations, at least in terms of internal consistency, reliability, and factor structure.

The W-BQ12 has shown good psychometric properties in a sample of people with macular disease [8] and growth hormone deficiency [7], including evidence of sensitivity to change [7], suggesting its usefulness as a generic measure of well-being. Furthermore, there is now evidence that the W-BQ12 is just as useful as the original W-BQ22. An evaluation of both versions of the W-BQ in multinational randomised-controlled trials of a new longer-acting insulin demonstrated that both versions were just as sensitive in detecting significant differences across time and between treatment groups [18].

Several limitations of this study should be noted. First, we did not include similar measures of affect for the purpose of providing further evidence of construct validity. However, no suitable measures were available in Japanese. Secondly, one third of our sample consisted of patients belonging to the Tenri-kyo religion, who might not be representative of the population of people with diabetes in Japan. However, the results from subgroup analysis indicate that there were no differences in the factor structure or reliability between the Tenri-kyo patients and those with other religious affiliations. Thirdly, we have used traditional psychometric analyses and did not use newer psychometric methods such as Rasch analyses [20], or structural equation modeling [21] to confirm the unidimensionality of the constructs being measured or to examine differential item functioning among the various subgroups. However, other investigators have since used confirmatory factor analysis to confirm the structure of the Japanese W-BQ12 in a Dutch sample [16].

## Conclusion

As the psychometric properties of instruments are sample dependent and cannot be established in a single study [22], further evaluations of the Japanese W-BQ12 are necessary, in particular to establish further its responsiveness, and its sensitivity to change across time with changes in treatment. The findings reported here demonstrate that the Japanese W-BQ12 has good evidence from a substantial sample of people with diabetes for the internal reliability of the three subscales and the total General Well-being scale, structural validity and preliminary evidence of construct validity. Thus the Japanese W-BQ12 is suitable for use with people with diabetes in Japan.

## Authors' contributions

AR translated the W-BQ22 into Japanese, carried out pilot-testing of the questionnaire, performed additional psychometric analyses and drafted the manuscript. CB conceived of the study, and participated in its design and coordination, helped to interpret the analyses and determine scale content and contributed to manuscript preparation. SB performed psychometric analyses and drafted a preliminary report. HI participated in the linguistic validation work and contributed to the design and coordination of the study.

## Access to the W-BQ12

For access to the W-BQ12 in any of its translations and associated user guidelines the copyright holder, Professor Clare Bradley can be contacted at c.bradley@rhul.ac.uk.
